# Reference guide for the diagnosis of adult primary immune thrombocytopenia, 2023 edition

**DOI:** 10.1007/s12185-023-03672-1

**Published:** 2023-11-13

**Authors:** Hirokazu Kashiwagi, Masataka Kuwana, Mitsuru Murata, Naoki Shimada, Toshiro Takafuta, Jun Yamanouchi, Hisashi Kato, Takaaki Hato, Yoshiaki Tomiyama, Committee for Reference Guide for diagnosis of adult ITP Research Team for Coagulopathy and Other Intractable Diseases supported by the Ministry of Health, Labor and Welfare Science Research Grant Project: Research on Health Care Policy for Intractable Diseases

**Affiliations:** 1 Department of Blood Transfusion, Osaka University Hospital, Suita, Osaka 565-0871, 2-15, Yamadaoka Japan; 2 Department of Allergy and Rheumatology, Nippon Medical School Graduate School of Medicine, Tokyo, Japan; 3 Center for Clinical Medical Research, International University of Health and Welfare, Ohtawara, Tochigi, Japan; 4 Center for Basic Medical Research, International University of Health and Welfare, Ohtawara, Tochigi, Japan; 5 Department of Internal Medicine, Hiroshima City Funairi Citizens Hospital, Hiroshima, Hiroshima, Japan; 6 Division of Blood Transfusion and Cell Therapy, Ehime University Hospital, Toon, Ehime, Japan; 7 Department of Hematology and Oncology, Graduate School of Medicine, Osaka University, Suita, Osaka, Japan; 8 Japanese Red Cross Ehime Blood Center, Matsuyama, Ehime, Japan

**Keywords:** Immune thrombocytopenia, Diagnosis, Thrombopoietin, Immature platelet fraction

## Abstract

Primary immune thrombocytopenia (ITP) is an autoimmune disorder characterized by isolated thrombocytopenia due to accelerated platelet destruction and impaired platelet production. Diagnosis of ITP is still challenging because ITP has been diagnosed by exclusion. Exclusion of thrombocytopenia due to bone marrow failure is especially important in Japan because of high prevalence of aplastic anemia compared to Western countries. Hence, we propose a new diagnostic criteria involving the measurement of plasma thrombopoietin (TPO) levels and percentage of immature platelet fraction (RP% or IPF%); 1) isolated thrombocytopenia with no morphological evidence of dysplasia in any blood cell type in a blood smear, 2) normal or slightly increased plasma TPO level (< cutoff), 3) elevated RP% or IPF% (> upper limit of normal), and 4) absence of other conditions that potentially cause thrombocytopenia including secondary ITP. A diagnosis of ITP is made if conditions 1-4 are all met. Cases in which criterion 2 or 3 is not met or unavailable are defined as “possible ITP,” and diagnosis of ITP can be made mainly by typical clinical course. These new criteria enable us to clearly differentiate ITP from aplastic anemia and other forms of hypoplastic thrombocytopenia and can be highly useful in clinical practice for avoiding unnecessary bone marrow examination as well as for appropriate selection of treatments.

## Introduction

In Japan, primary immune thrombocytopenia (ITP) has conventionally been diagnosed according to the diagnostic criteria revised in 1990 by the former Ministry of Health and Welfare Research Committee. These diagnostic criteria primarily involve diagnosis by exclusion. Certain aspects of these criteria warranted revision: for example, platelet-associated IgG (PAIgG), as used here, was of little diagnostic value due to a lack of specificity [1]; while bone marrow examination, which is considered necessary, does not yield findings specific to ITP [2]. Most notably, aplastic anemia can be misdiagnosed as ITP when anemia is preceded by thrombocytopenia in the early stage, and bone marrow examination does not always reveal hypoplasia depending on the puncture site [3]. Roughly 10% of cases diagnosed as refractory ITP are bone marrow failure [4], and differentiating ITP from aplastic anemia is considered more important in Japan, where the prevalence of aplastic anemia is markedly higher that in the West [5]. Our research group has tackled this issue for many years. In 2006, we proposed diagnostic criteria incorporating anti-GPIIb/IIIa antibody-producing B cells (ELISPOT), platelet-associated (PA) anti-GPIIb/IIIa antibodies, plasma thrombopoietin (TPO) levels, and percentage of reticulated platelets (RP%) [6]. Here, we have proposed new diagnostic criteria involving the measurement of plasma TPO levels and percentage of immature platelet fraction, which includes RP%. These biomarkers reflect the pathology of ITP, which is characterized by a shortened platelet lifespan associated with platelet destruction and relatively maintained platelet production [7]. While our diagnostic criteria resemble the previous criteria in that they emphasize the exclusion of many other diseases, these new criteria enable clear differentiation of ITP from aplastic anemia and other forms of hypoplastic thrombocytopenia. Therefore, our new diagnostic criteria can be highly useful in clinical practice for avoiding unnecessary bone marrow examination as well as appropriate selection of treatments.

## Diagnostic criteria (Table 1)

**Table 1:** Diagnostic criteria

1. ALL of the following^*1^
Thrombocytopenia (< 100,000/μL)
Absence of anemia (excluding anemia due to bleeding and/or iron deficiency)
Normal leukocyte count (but may present with mild abnormal leukocyte count)
No morphological evidence of dysplasia in any blood cell type in a blood smear
2. Normal or slightly increased plasma TPO level (< cutoff)^*2^
3. Elevated percentage of immature platelet fraction (RP% or IPF%; > ULN)
4. Absence of other conditions that potentially cause thrombocytopenia including secondary ITP^⋆3^

*ULN* upper limit of normal

**A diagnosis of ITP is made if conditions 1–4 above are all met**

**Cases in which criterion 2 or 3 is not met or unavailable are defined as “possible ITP”. A diagnosis of ITP can be made if the patient is a glycoprotein (GP)-specific platelet-antibody positive, increased GP-specific platelet antibody-producing B cells, or typical clinical course**

^1^Bone marrow examination is strongly recommended to rule out other diseases if peripheral blood demonstrates any of the following findings: leukocyte count < 3000 or ≥ 10,000/μL, MCV ≥ 110, neutrophils < 30% or lymphocytes ≥ 50%, presence of immature leukocytes

^2^The cutoff is 300 pg/mL with a R&D Systems ELISA Kit and 70 pg/mL with a MBL kit (TPO-CLEIA)

^3^Includes drug-related thrombocytopenia, radiation injury, aplastic anemia, myelodysplastic syndrome (MDS), paroxysmal nocturnal hemoglobinuria (PNH), systemic lupus erythematosus (SLE), leukemia, malignant lymphoma, bone marrow metastasis of cancer cells, disseminated intravascular coagulation (DIC), thrombotic thrombocytopenic purpura (TTP), hypersplenism, megaloblastic anemia, sepsis, infections (tuberculosis, etc.), sarcoidosis, and huge hemangioma such as Kasabach-Merritt syndrome. Congenital diseases involving thrombocytopenia include Bernard–Soulier syndrome, Wiskott–Aldrich syndrome, MYH9-related disease and Upshaw–Schulman syndrome

### Detailed explanations of diagnostic criteria

#### ALL of the following

① Thrombocytopenia (< 100,000/μL).
② Absence of anemia (excluding anemia due to bleeding and iron deficiency anemia)
③ Normal leukocyte count (but may present with slightly abnormal leukocyte count)
④ No morphological evidence of dysplasia in any blood cell type in a blood smear

ITP does not demonstrate any hematopoietic abnormalities other than thrombocytopenia. However, microcytic anemia due to bleeding and/or iron deficiency is frequently observed. In contrast, macrocytic anemia (MCV ≥ 110) is unusual in ITP and requires bone marrow examination to exclude other diseases, such as myelodysplastic syndrome (MDS). In addition, while mild leukopenia and leukocytosis are not uncommon in ITP, bone marrow examination is strongly recommended in the event of any of the following: leukocyte count < 3000/μL or ≥ 10,000/μL, immature or morphological abnormal leukocytes in a peripheral blood smear, reduced neutrophil levels, or elevated lymphocyte levels.

#### Normal or slightly increased plasma TPO level

TPO is constantly produced primarily by the liver, while plasma TPO levels are regulated by its binding and clearance via TPO receptors (c-MPL) expressed on platelets and megakaryocytes [8]. Thus, plasma TPO levels are inversely proportional to the rate of platelet production, and plasma TPO levels dramatically increase in hypoplastic thrombocytopenia such as aplastic anemia. In sharp contrast, in ITP, both enhanced platelet destruction accelerating the disappearance of platelet-bound TPO from plasma and the relatively preserved number of megakaryocytes keep plasma TPO levels to remain normal to slightly elevated despite thrombocytopenia. A recent study in mice has revealed a novel mechanism of TPO regulation in which desialylated senescent platelets are phagocytosed via hepatocyte Ashwell–Morell receptors, promoting the production of TPO [9]. While anti-GPIbα antibodies can promote desialylation [10], the presence of anti-GPIbα antibodies in ITP has not been found to affect plasma TPO levels in humans [11]. Thus, this novel mechanism of TPO regulation remains elusive in humans.

As shown by many studies, measurement of plasma TPO level is incredibly useful for differentiating between ITP and hypoplastic thrombocytopenia [12–30]. However, in chronic hepatitis, while TPO levels are normal to slightly elevated, the progression of fibrosis causes TPO levels to decrease [22, 30–32]. While thrombotic thrombocytopenic purpura (TTP) and antiphospholipid syndrome (APS) are similar to ITP in terms of plasma TPO levels [12, 19], plasma TPO levels are elevated in severe cases of disseminated intravascular coagulation [19, 33–35]. They are also normal to slightly elevated in congenital thrombocytopenia [24, 27]. Plasma TPO levels range from normal to high in MDS, as discussed in detail later.

Most studies have used the R&D Systems ELISA Kit (Quantikine, Minneapolis, MN, USA, for research use only) to measure plasma TPO levels with normal ranges of 40 − 120 pg/mL [20, 23, 27, 36, 37]. In our previous studies that used the R&D Systems kit, the mean level of plasma TPO in healthy individuals was 83.9 ± 11.7 (50‒126) pg/ml, with levels ≤ 142 pg/mL (mean + 5 SD) considered normal [7]. In differentiation between ITP and non-ITP, 300 pg/mL is the most sensitive and specific value; therefore, with the R&D Systems kit, 300 pg/mL is used as the cutoff for plasma TPO level [6, 37]. A chemiluminescent enzyme immunoassay (CLEIA)-based TPO measurement kit (TPO-CLEIA, MBL, Japan) for clinical diagnostic use has recently been developed in Japan; with this kit, a TPO level of 300 pg/mL with the R&D Systems kit is equivalent to 70 pg/mL with the TPO-CLEIA, which is therefore used as the cutoff of this TPO-CLEIA kit [29].

#### Elevated immature platelet fraction (RP% or IPF%)

In ITP, immune mechanisms enhance platelet destruction and shorten platelet lifespan, causing the percentage of immature platelets to increase. Patients with ITP also exhibit increased larger immature platelets compared to healthy subjects [38]. The standard method to detect immature platelets is to identify RNA-rich platelets in flow cytometry using thiazole orange as reticulated platelets (RP), as with the detection of reticulocytes [39, 40]. However, RP measurement is complicated and not standardized yet, making it unsuitable as a laboratory test. A recently developed method enables measurement of immature platelet fraction (IPF) with an automated hemocytometer (Sysmex, Japan). However, this method has several drawbacks: measurement in terms of IPF may not necessarily be identical to RP; measurements were strongly affected by schistocytes, and data obtained from severe thrombocytopenia were less reliable due to extremely small platelet measurement counts, especially in older devices (Sysmex XE series) [41]. The new XN-Series and XR-Series, which use dedicated staining reagents and a dedicated measurement channel, were developed to overcome these drawbacks, and have sensitivity and specificity similar to those for RP% [41–44].

As reported by many studies, elevated RP% and IPF% have been observed in ITP [6, 22, 23, 30, 36, 41, 45–67]. However, increased RP% and IPF% are not specific to ITP; rather, they are also observed in DIC, TTP, and other forms of thrombocytopenia caused primarily by enhanced platelet consumption [66, 67]. Values of IPF% vary in MDS and other hematologic malignancies. It is worth noting that IPF% is likely high in MDS with poor prognostic chromosomal abnormalities including chromosome 7 [53, 55, 56, 68, 69]. In addition, IPF% is more greatly affected by platelet size than RP%. Therefore, IPF% is often markedly elevated in MYH9-related disease, Bernard–Soulier syndrome, and other congenital macrothrombocytopenia [61, 70]. When IPF% is markedly elevated in comparison to platelet count (platelet count ≥ 50,000/μL and IPF ≥ 10%), the possibilities of MDS and congenital macrothrombocytopenia must be considered.

Because normal values of RP% and IPF% have not been standardized, values exceeding the upper limit of normal (ULN) are considered elevated.

#### Other diseases that can cause thrombocytopenia can be ruled out.

Major diseases to differentiate from ITP will be discussed later.

#### Supplemental explanations for diagnostic criteria

- A diagnosis of ITP is made if conditions 1 - 4 above are all met
- Cases in which criterion 2 or 3 is not met or unavailable are defined as “possible ITP”. A diagnosis of ITP can be made if the patient is glycoprotein (GP)-specific platelet-antibody positive, the patient demonstrates increased GP-specific antiplatelet antibody-producing B cells, or a typical clinical course

A typical case of ITP meets all conditions 1–4. In particular, a combination of plasma TPO level and IPF% can distinguish ITP from hypoplastic thrombocytopenia with high sensitivity and specificity [6, 22, 23, 30, 47]. However, plasma TPO levels occasionally exceed standard levels, and plasma TPO levels in acute phase of ITP may be higher as compared in chronic ITP [71]. We recommend to repeat measurement of plasma TPO levels with high TPO levels in typical ITP cases. Regarding elevated immature platelet fraction (RP% or IPF%), many studies have reported that RP% and IPF% are elevated in 60–95% and ≥ 80% of ITP cases, respectively. However, some ITP patients did not show elevation of these markers [6, 23, 41, 50, 51, 56, 57, 62]. Therefore, cases that do not meet the criteria for either plasma TPO level or elevated percentage of immature platelet fraction are diagnosed as “possible ITP”. When possible, a diagnosis of ITP should be made based on GP-specific platelet antibodies or a GP-specific antiplatelet antibody-producing B cell (ELISPOT) test [6]. If these tests are unavailable, ITP can be diagnosed based on clinical course, particularly the effects of corticosteroid therapy and/or high-dose immunoglobulin therapy.

## Diagnostic procedure (Fig. 1)

**Fig. 1:**
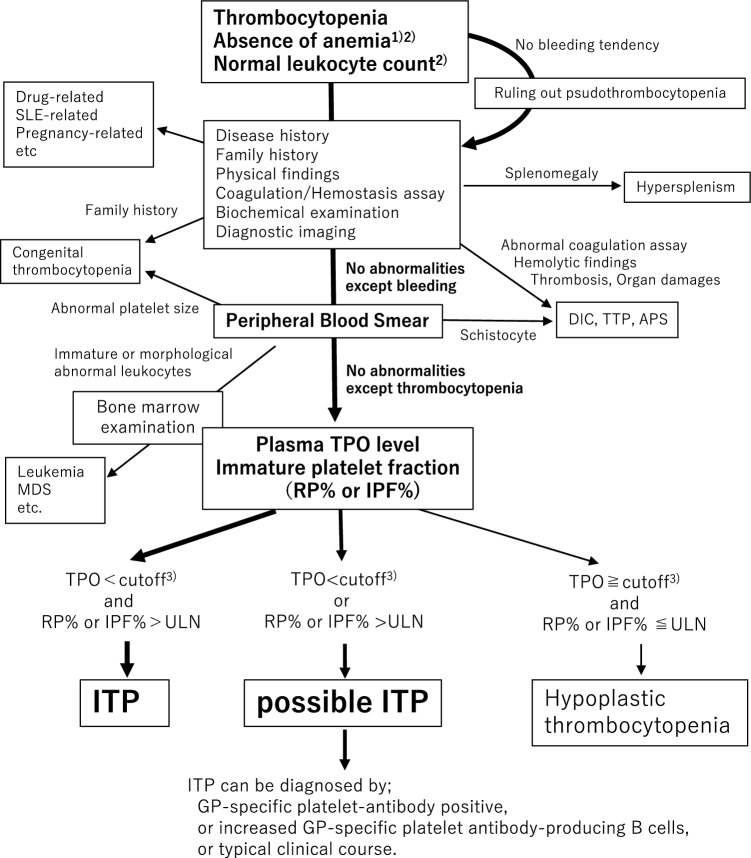
Flowchart of ITP diagnosis Anemia due to bleeding and iron deficiency anemia should be excluded. Bone marrow examination is strongly recommneded in the event of any of the following: leukocyte count < 3000/μL or ≥ 10,000/μL, immature or morphological abnormal leukocytes in a peripheral blood smear, reduced neutrophil levels, or elevated lymphocyte levels. Cutoff values are 300 pg/ml with the R&D Systems kit and 70 pg/ml with TPO-CLEIA (MBL). *APS* antiphospholipid syndrome, *DIC* disseminated intravascular coagulation, *GP* platelet glycoprotein, *MDS* myelodysplastic syndrome, *SLE* systemic lupus erythematosus, *TPO* thrombopoietin, *TTP* thrombotic thrombocytopenia purpura, ULN: upper limit of normal

Figure 1 shows the flowchart for diagnosing ITP. We have introduced plasma TPO level and immature platelet fraction in the diagnostic criteria. While these biomarkers reflect the pathology of ITP, they do not constitute a specific test for ITP exclusively. Therefore, it is crucial to carefully differentiate ITP from other diseases that cause thrombocytopenia.

The key points in the diagnostic procedure are described below.

1. Ruling out pseudothrombocytopenia

Pseudothrombocytopenia is first ruled out when there are no abnormalities other than thrombocytopenia and no bleeding symptoms. Pseudothrombocytopenia is a state in which platelets agglutinate in vitro, causing the hemocytometer to yield a low platelet count. While pseudothrombocytopenia is considered to occur most commonly due to antibodies recognizing GPIIb/IIIa that has undergone structural alteration in the presence of EDTA [72, 73], platelets can also agglutinate in the presence of citrate and blood collection with heparin, though the detailed mechanism of agglutination has not yet been determined [74]. It is important to confirm the presence of platelet aggregates in a peripheral blood smear. When possible, assessing platelet count immediately following blood collection without any anticoagulant can yield a more accurate result. Also, although extremely rare, cold agglutinins are reported to cause pseudothrombocytopenia [75].

2) Basic assessment
  Disease history
  Family history
  Physical findings
  Complete blood count (CBC)
  Peripheral blood smear
  Biochemical examination

Diagnostic interviews should confirm the course of thrombocytopenia and bleeding symptoms, previous infections, comorbidities, medications used, and family history of thrombocytopenia. The nature of bleeding symptoms should be noted carefully. Purpura in ITP often typically range in size from petechiae to ecchymosis. Mucosal bleeding (epistaxis, gastrointestinal bleeding, hematuria, etc.) is frequently observed in more serious cases of thrombocytopenia, and fatal intracranial hemorrhage occurs in roughly 1% of adults and 0.4% of children with ITP [76]. Intra-articular bleeding, intramuscular bleeding, and other deep hemorrhages observed in conditions such as hemophilia are rare. Adults with ITP often do not demonstrate bleeding symptoms.

As for peripheral blood findings, bone marrow examination is strongly recommended to rule out other diseases in the following cases: anemia other than iron deficiency or hemolytic anemia is observed, leukocyte count is abnormal (< 3000/μL or ≥ 10,000/μL), a peripheral blood smear reveals the presence of immature leukocytes or morphological defects, neutrophil count is decreased, or lymphocyte count is increased. In a biochemical examination, liver dysfunction, increased LD, and inflammatory findings require particular attention. While ITP shows an increased percentage of large platelets, congenital macrothrombocytopenia should be considered in the event of markedly increased large to giant platelets. Ruling out SLE, which frequently occurs in secondary ITP, requires consideration of antinuclear antibodies and complements as well as examination of physical abnormalities such as fever, arthralgia/arthritis, and findings in the skin (erythema, etc.), kidneys, lungs, heart, and central nervous system [77].

3) Tests to perform next
  Coagulation/hemostasis assay
  Confirmation of hemolysis findings (bilirubin, LD, haptoglobin, and schistocytes)
  Diagnostic imaging
  Helicobacter pylori test
  HIV, HBV, and HCV

When a routine coagulation/hemostasis assay (PT, aPTT, fibrinogen, FDP) reveals abnormalities, DIC and other diseases should be excluded. When hemolysis findings (elevated bilirubin, elevated LD, decreased haptoglobin) are observed, a Coombs test should be performed, and the possibility of Evans syndrome should be considered. When a Coombs test is negative, or in the event of neurological symptoms, renal dysfunction, or schistocytes, ADAMTS13 activity should be measured to rule out TTP. Imaging should be considered to assess for splenomegaly. Testing should also be performed for *H. pylori*, HIV, HBV, and HCV, all of which are potentially associated with ITP.

4) Testing is performed when evident abnormalities other than thrombocytopenia are not observed
  Plasma TPO levels
  Immature platelet fraction (RP% or IPF%)

When the above tests do not reveal any evident abnormalities other than thrombocytopenia, plasma TPO levels and immature platelet fraction (RP% or IPF%) are measured, and ITP is diagnosed based on the criteria described above.

## Diseases to differentiate from ITP and their characteristics

1. Congenital (hereditary) thrombocytopenia

Congenital thrombocytopenia is a rare disease but is reported to account for roughly 15% of diagnoses of refractory ITP [4]. Congenital thrombocytopenia not only poses a risk of unnecessary treatment such as splenectomy due to misdiagnosis as ITP but can also involve nephritis, deafness, and cataract in MYH9-related disease as well as leukemia in FPD-AML; therefore, careful differentiation of ITP and congenital thrombocytopenia is required [78]. In diagnosing ITP, it is important to confirm the family history and thoroughly review peripheral blood smear findings. Congenital thrombocytopenia can be classified by platelet size: MYH9-related disease, Bernard–Soulier syndrome, and GPIIb/IIIa-induced thrombocytopenia, which are relatively frequent, are classified as congenital “macro” thrombocytopenia. MYH9-related disease presents with ≥ 50% giant platelets and characteristic leukocyte inclusion bodies [79]. In congenital macrothrombocytopenia, IPF% is often markedly high compared to platelet count, a characteristic that facilitates differentiation from ITP [61, 70].

2) Drug-related thrombocytopenia

Drug-related thrombocytopenia is broadly divided into two types: non-immune-mediated, which is typified by anticancer drug induced myelosuppression, and immune-mediated. More than 300 drugs have been reported to induce immune thrombocytopenia [80]. Drug-related immune thrombocytopenia typically manifests 5 − 10 days following drug administration and not uncommonly presents with a dramatically reduced platelet count (≤ 20,000/μL). Known mechanisms involved in drug-related immune thrombocytopenia include the following: i) hapten-dependent antibodies: drug binds to platelet membranes as haptens and react with antibodies (penicillin, cephalosporins); (ii) quinine-type antibodies: drug binds to antibody Fab and membrane GP, enhancing antibody affinity and binding to platelet GP (quinine, vancomycin, rifampicin, etc.); (iii) autoantibody induction: drug induces the formation of platelet autoantibody that reacts in a non-drug-dependent fashion (procarbazine, gold salts, etc.); (iv) immune complexes: drug/PF4 immune complex activates platelets via FcγRIIa (heparin) [81].

3) Gestational thrombocytopenia

Thrombocytopenia is observed in approximately 10% of all pregnant women, roughly 70% of those cases being gestational thrombocytopenia. Although its pathology is unknown, it typically occurs in the second to third trimester; thrombocytopenia is mild, with platelet count typically ≥ 70,000/μL. Gestational thrombocytopenia spontaneously remits 1 − 2 months after delivery without triggering fetal or neonatal thrombocytopenia. Since there is no clear method for distinguishing between gestational thrombocytopenia and ITP in pregnancy, it is important to carefully observe the course of thrombocytopenia when it first appears during pregnancy. Caution is required when gestational thrombocytopenia involves hemolysis findings. Although rare, congenital TTP (Upshaw–Schulman syndrome) should be considered as a possibility, and ADAMTS13 activity should be measured [82]. Refer to other guidelines regarding diseases to consider in relation to thrombocytopenia during pregnancy [83].

4) Aplastic anemia

Aplastic anemia is characterized by marrow hypoplasia from immune attack on hematopoietic cells of unknown etiology, resulting in pancytopenia [84]. During its course, some patients who were initially diagnosed as aplastic anemia develop MDS or acute myeloid leukemia [85]; and bone marrow aspiration and biopsy are needed to assess clonal evolution. However, chromosomal abnormalities are also detected in some patients with aplastic anemia [86]. Abnormalities in chromosome 7 (monosomy 7, etc.) are known to be associated with a high risk of progression to MDS/acute myeloid leukemia and poor prognoses [87, 88]. In contrast, some abnormalities such as trisomy 8 [89, 90], del(13q) [91, 92], and loss of heterozygosity in the short arm of chromosome 6 (6pLOH) [93] are unrelated to poor prognosis. While bone marrow in aplastic anemia is characterized by hypoplastic bone marrow with fatty replacement, the residual patchy hematopoiesis may affect accurate assessment of hematopoiesis by aspiration and/or biopsy. Therefore, magnetic resonance imaging (MRI) is useful to assess a homogeneous fatty bone marrow observed in aplastic anemia [94].

In some cases of aplastic anemia, isolated thrombocytopenia can be observed in its early stage, making it difficult to differentiate from ITP, and roughly 10% of patients with refractory ITP are misdiagnosis of bone marrow failure including aplastic anemia [4]. In bone marrow examination, the number of megakaryocytes is an important point for differentiation. Although there are no specific findings in the bone marrow with ITP, the number of megakaryocytes is generally normal to increased. In contrast, the number of megakaryocytes is decreased in aplastic anemia. Immature platelet fraction and plasma TPO level, which we have proposed here as diagnostic criteria for ITP, are highly effective for the differential diagnosis. Significant differences between aplastic anemia and ITP are the lack of an increased immature platelet fraction and a markedly increased plasma TPO level in the case of aplastic anemia [12–18, 20–24, 26–29]. Increased PNH clones, which are detected in 30% − 60% of cases of aplastic anemia, may also be useful in the differential diagnosis between ITP and aplastic anemia [95].

5) MDS

MDS occurs most frequently among elderly adults. Among patients with MDS, 80– 85% present with anemia, 30–65% present with thrombocytopenia, and 40–50% present with neutropenia [96]. Although MDS often involves reductions in at least two types of blood cells, 1–10% of patients demonstrate only thrombocytopenia, hindering differentiation from ITP [97–101]. Patients with MDS sometimes demonstrate markedly increased IPF%. Such cases often demonstrate markedly heterogeneous platelet size and chromosomal abnormalities including chromosome 7 with poor prognoses [68]. Plasma TPO levels vary greatly from normal to high [22, 30, 31]. Notably, patients with markedly elevated plasma TPO levels often have increased PNH-type cells and respond to ciclosporin [102, 103], a pathology surmised to resemble that of aplastic anemia. As illustrated above, MDS and ITP are difficult to differentiate based on immature platelet fraction and plasma TPO level. Proactive bone marrow examination is recommended for differentiating ITP from MDS in the following instances: high MCV [104], abnormal leukocyte count, blood cell morphological defects, the appearance of immature leukocytes, or abnormal neutrophil–lymphocyte ratio, or refractory cases of ITP.

6) Thrombocytopenia with increased thrombus formation: DIC, TTP, APS, etc

In thrombocytopenic diseases such as DIC, TTP, and APS, increased thrombus formation may induce enhanced platelet consumption, in turn causing thrombocytopenia. While TTP dominantly present symptoms associated with thrombus formation, TTP occasionally exhibits bleeding tendency with severe thrombocytopenia resembling ITP. These diseases often demonstrate the same trends observed for ITP in immature platelet fraction and plasma TPO level, which have been adopted in this guideline as diagnostic criteria for ITP [12, 19, 33–35, 66, 67]. For DIC, coagulation and fibrinolytic system testing, namely FDP, fibrinogen, and prothrombin time, are useful for diagnosis. We recommend differentiation of TTP from ITP based on confirmation of schistocytes in a peripheral blood smear and hemolysis findings (elevated LD, decreased haptoglobin, etc.) and consideration of clinical findings such as nephropathy and neurologic symptoms. Differentiation of TTP from ITP via measurement of ADAMTS13 activity is particularly important when hemolysis findings are observed. As for APS, in the event of thrombosis, habitual abortion, or other clinical findings indicative of APS, an antiphospholipid antibody test should be performed. For all the above diseases, diagnostic criteria and therapeutic guidelines have been published; refer to these when differentiating these diseases from ITP [105–108].

7) Secondary ITP

Underling diseases of secondary ITP include connective tissue diseases (SLE, mixed connective tissue disease, Sjögren's syndrome, etc.), lymphoproliferative disease, chronic hepatitis, and HIV infection. Primary and secondary ITP share the same pathogenic process, mediated through immune dysregulation consisting primarily of anti-platelet autoantibodies. This hinders differentiation based on diagnostic criteria 1 − 3 [109–111]. Therefore, exclusion of secondary ITP is based on detailed interviews, physical findings, and test results specific to underlying diseases (e.g. virus tests). SLE often involves not only thrombocytopenia but also decreased neutrophil, lymphocyte, and erythrocyte counts, with the frequency of lymphocytopenia serving as useful information. Pancytopenia is also found in patients with cirrhosis when portal hypertension is complicated. Although the thrombocytopenia observed in SLE is mediated mainly by secondary ITP, other pathogenic mechanisms, such as amegakaryocytic thrombocytopenia, DIC, thrombotic microangiopathy, APS, and non-specific platelet destruction associated with immune complexes account for thrombocytopenia in SLE.

## Other tests associated with ITP diagnosis

- Detection of antiplatelet autoantibodies

Although PAIgG has high sensitivity in ITP diagnosis, it lacks specificity; a positive result for PAIgG cannot be used as a maker for diagnosing ITP [112, 113]. One conceivable reason for this poor disease specificity is that PAIgG includes not only platelet autoantibodies but also non-specific IgG attached to platelets. To overcome this flaw, methods have been developed to detect platelet antibodies that recognize platelet membrane glycoproteins (GP); such methods include monoclonal antibody-specific immobilization of platelet antigen (MAIPA) [114] and modified antigen capture ELISA (MACE) [115], which involve capturing target antigens with monoclonal antibodies and detecting autoantibodies against them with ELISA. The most common target antigens are GPIIb/IIIa and GPIb/IX; antibodies against GPIIb/IIIa alone, GPIb/IX alone, and both are detected in 68%, 18%, and 15% of ITP patients, respectively [116, 117]. Although the sensitivity of these GP-specific platelet antibodies in ITP diagnosis remains 49−66%, their specificity is 80−90%, meaning that a positive result enables a proactive diagnosis of ITP [117–119]. Furthermore, the detection of antibodies or the number of target antigens may be related to the remission and seriousness of ITP [120]. However, measurement of these GP-specific platelet antibodies is currently only at the laboratory level.

b) Detection of anti-platelet antibody-producing B cells (ELISPOT)

In circulation, GP-specific platelet antibodies are primarily bound to platelet membrane surfaces, requiring techniques to lyse platelets for detection of these antibodies. Therefore, an assay using the enzyme-linked immunospot (ELISPOT) principle has been proposed to detect anti-platelet antibody-producing B cells [121, 122]. Specifically, mononuclear cells isolated from the patient’s peripheral blood are cultured on the solid-phased membranes of GP (GPIIb/IIIa, GPIb, etc.), followed by detection of anti-GP antibodies. While ELISPOT is useful for differentiating ITP from MDS and aplastic anemia, the method is not widespread as a standard laboratory test because the assay requires immediate separation after blood drawing. Assays using this principle can be developed into kits, and measurements can be outsourced to testing centers.

c) Bone marrow examination

The primary purpose of bone marrow examination in ITP diagnosis is to rule out diseases other than ITP. Although bone marrow examination has indisputably played a role in differentiating ITP from diseases such as MDS and aplastic anemia, recent studies have claimed that bone marrow examination is unnecessary in this regard [123–126]. Guidelines and consensus reports have also stressed that bone marrow examination is unnecessary [127, 128]. However, bone marrow examination has been conventional until now, with 50% of examinations reported to have been unnecessary [129]. There are no bone marrow findings unique to ITP; increased megakaryocytes, which are considered characteristic of ITP, are observed in only 25% of cases; and the sensitivity and specificity of bone marrow examination in ITP diagnosis are considered to be 24% and 90%, respectively [130]. Bone marrow examination may be necessary for circumstances such as the following: abnormal hematological findings other than thrombocytopenia (including smear findings), splenomegaly, lymphadenopathy, advanced age (≥60 or ≥65 years), treatment refractoriness, and planned use of TPO receptor agonists [131]. Although criteria for assessing abnormal hematological findings are lacking in evidence, potential criteria include the following: anemia, morphological abnormalities of blood cells, the appearance of immature leukocytes, abnormal leukocyte count, abnormal neutrophil ratio or lymphocyte ratio, and high MCV. The present diagnosis reference guide also recommends bone marrow examination when the above findings are observed. In actual bone marrow examinations, factors such as nucleated cell count, megakaryocyte count, morphological abnormalities in all three lineages of blood cells, B cell and T cell clonality, and chromosomal abnormalities should be examined. At present, the indication for bone marrow examination might be difficult to determine based on the proposed diagnostic markers (immature platelet fraction and plasma TPO level). However, we assume that our proposed diagnostic markers would contribute to the more accurate diagnosis of ITP, which would make bone marrow examination less frequent.

d) Other tests (Ig quantitation, antiphospholipid antibodies, thyroid-related tests, antinuclear antibodies, and DAT)

Due to potential comorbid common variable immunodeficiency, IgG, A, and M should be quantitated as part of testing for ITP [128]. Results for anti-cardiolipin antibodies, lupus anticoagulant, and other antiphospholipid antibodies are positive in 25− 30% of patients with ITP [132–134]. Immunoglobulins should be quantitated before using TPO receptor agonists or similar agents to assess the risk of thrombosis. A positive result for antinuclear antibodies suggests the possibility of chronic or secondary ITP [135, 136]. Tests for antithyroid antibodies and thyroid dysfunction should also be performed because these are often present in patients with ITP [137]. The possibility of comorbid autoimmune hemolytic anemia (Evans syndrome) should also be considered in direct Coombs testing.

## Conclusion

Measurement of the combination of immature platelet fraction and plasma TPO level in appropriate cases enable clear differentiation of ITP from hypoplastic thrombocytopenia, which has sometimes been difficult in the past. However, it remains crucial to rule out many other diseases by several screening examinations. Further evaluation of the validity of our diagnostic criteria is necessary.
